# circCRAMP1L is a novel biomarker of preeclampsia risk and may play a role in preeclampsia pathogenesis via regulation of the MSP/RON axis in trophoblasts

**DOI:** 10.1186/s12884-020-03345-5

**Published:** 2020-10-27

**Authors:** Yonggang Zhang, Hongling Yang, Yipeng Zhang, Junzhu Shi, Ronggui Chen

**Affiliations:** 1 Department of Clinical Laboratory, Shenzhen Longhua District Central Hospital, Guangdong Medical University, Shenzhen, 518110 Guangdong China; 2 Department of Clinical Laboratory, Guangzhou Women and Children’s Medical Centre, Guangzhou Medical University, 9, Jinsui Road, Guangzhou, 510623 China

**Keywords:** circCRAMP1L, MSP/RON, Preeclampsia

## Abstract

**Background:**

Preeclampsia is a severe disease in pregnant women, which is primarily managed by early screening and prevention. Circular RNAs (circRNAs) have increasingly been shown to be important biological regulators involved in numerous diseases. Further, increasing evidence has demonstrated that circRNAs can be used as diagnostic biomarkers. This study was conducted to evaluate the potential of circCRAMP1L, previously identified to be downregulated in preeclampsia, as a novel biomarker for predicting the development of preeclampsia.

**Methods:**

We measured the expression of circCRAMP1L, which is reportedly relevant to trophoblast physiology, in plasma samples from 64 patients with preeclampsia and 64 age-, gestational age-, and body mass index-matched healthy pregnant women by qRT-PCR. MTT proliferation and transwell invasion assays revealed the biological role of circCRAMP1L in preeclampsia pathogenesis. RNA immunoprecipitation and dual-luciferase reporter assays clarified the mechanism underlying the biological function of circCRAMP1L in TEV-1 cells.

**Results:**

circCRAMP1L circulating levels were significantly lower in patients with preeclampsia (2.66 ± 0.82, △Ct value) than in healthy pregnant women (3.95 ± 0.67, △Ct value, *p* <  0.001). The area under the receiver operating characteristic curve for circCRAMP1L was 0.813. Univariate and multivariate analyses identified circCRAMP1L as an independent predictor of preeclampsia. Furthermore, when circCRAMP1L was utilised in combination with its target protein macrophage stimulating protein (MSP), the predictive performance increased, with an area under the receiver operating characteristic curve of 0.928 (95% CI 0.882–0.974), 80.0% sensitivity, and 80.0% specificity. The in vitro results indicated that circCRAMP1L regulates cell proliferation, and invasion via MSP and RON proteins. We investigated the molecular mechanisms of these effects. In vitro, relative to the control group, circCRAMP1L overexpression significantly enhanced cell proliferation; furthermore, trophoblast cell invasion increased proportionally with circCRAMP1L expression. RNA immunoprecipitation and luciferase reporter gene illustrated that circCRAMP1L participated in regulation of trophoblast cell by regulating MSP.

**Conclusion:**

Reduced plasma levels of circCRAMP1L may be associated with an increased risk of preeclampsia, and circCRAMP1L may be a novel biomarker of preeclampsia risk.

**Supplementary information:**

**Supplementary information** accompanies this paper at 10.1186/s12884-020-03345-5.

## Background

Preeclampsia (PE), a pregnancy-specific syndrome, has a worldwide incidence of approximately 2–8%. With economic and social development, maternal and paternal ages are increasing, leading to larger number of cases of PE [1, 2]. PE can threaten the short-term or long-term health of mothers and their offspring [3, 4]. However, methods for preventing and treating PE remain ineffective [5]. Therefore, studies are urgently needed to clarify the pathogenesis of PE, improve effective early interventions, reduce the risk of PE, and lower the mortality of pregnant women and foetuses.

Recent studies showed that dysplasia of the placenta is a key factor in the pathogenesis of PE [6]. The theory holds that obstruction of the trophoblast differentiation phenotype during placenta formation leads to inadequate invasion and poor spiral artery remodelling, resulting in vascular endothelial cell damage, hypertension, and urinary protein in patients [6, 7]. The main pathological manifestation of the placenta in patients with PE is that trophoblasts invade the endometrium too shallowly and only reach the decidual layer, providing experimental evidence for this theory [8].

In recent decades, the pathogenesis and prediction of PE have been widely evaluated by obstetric researchers [9]. The general international consensus is that early prevention and early intervention are the primary means of managing PE [10]; this requires effective prediction of the occurrence of PE. However, current prediction methods for PE remain limited and inefficient [5].

Circular RNA (circRNA) is a type of cyclic non-coding RNA. In contrast to traditional linear RNA, circRNA molecules have a closed ring structure that is not affected by RNA exonuclease [11]. These molecules are more stable, difficult to degrade, and exerts biological roles by directly regulating protein expression [12]. Numerous studies have reported that circRNA can affect cell proliferation, apoptosis, invasion, and metastasis [13, 14]. Previously, we and others found that circRNAs expressed in circulating blood and placenta tissue were related to PE [15, 16]. According to previous microarray analysis of circRNAs, circCRAMP1L was downregulated in PE blood. Through bioinformatics prediction and pre-experiments, we found that circCRAMP1L can interact with macrophage stimulating protein (MSP). This study was conducted to investigate the role of circCRAMP1L in PE and verify whether circCRAMP1L can be used as a biomarker for PE.

## Methods

We carried out a prospective investigation of circCRAMP1L expression levels in PE and control. In the study, we followed up the pregnant women until one week postpartum. Then, we picked out who was diagnosed as PE and matched control with the maternal age, gestational age, and body mass index.

### Patient and samples

The study was approved by the Medical Ethics Committees (permission number: 2018045) and written consent was acquired from enrolled subjects. All experimental steps follow the Helsinki declaration. PE and healthy control pregnancy or preterm labour (PTL) control samples were gathered from June 2017 to May 2019. A total of 1128 pregnant women were enrolled in the study. Inclusion criteria is that pregnancy women who were at 8–20 gestation weeks; exclusion conditions included trophoblastic disease, multiple pregnancies, individual taking medicine (particularly aspirin, antibiotics), alcohol intake, and diseases such as liver and kidney diseases.

Blood was collected and processed in accordance with the Standard Operating Procedures for Plasma Collection before 20th gestation weeks. Fresh peripheral venous blood samples (5 mL) were collected with EDTA and then centrifuged (3000 rpm for 10 min at 4 °C). The plasma was immediately separated and stored at − 80 °C until use. Thirty pairs of PE and PTL control placental samples were taken from combining quadrants of the maternal-foetal interface near the maternal side and stored in a liquid nitrogen tank after delivery.

### Fluorescent in situ hybridisation and immunohistochemistry

Fluorescent in situ hybridisation analysis was performed as previously described [17]. Briefly, tissue was seeded on a coverslip, fixed with paraformaldehyde, and incubated with formamide, saline-sodium citrate buffer, *Escherichia coli* transfer RNA, salmon sperm DNA, bovine serum albumin, and DIG-labelled probe (5′-AATGCAACAAAGCTGAATGAACTCATTCAGGTTGGGAGCTGAAGGT − 3′).

for circCRAMP1L junction. The probes for GAPDH were from a DIG Labelling Kit (MyLab Corporation, Beijing, China). Hybridisation was carried out in a humidified chamber. circCRAMP1L was detected with a Fluorescent In Situ Hybridisation Kit (RiboBio, Guangzhou, China), and the nuclei were counterstained with 4′, 6-diamidino-2-phenylindole.

The expression of the target protein in the placenta specimen was detected with immunohistochemical method as previously described [18]. Briefly, Paraffin section of placenta were incubated with primary antibodies MSP or RON (Abcam, Cambridge, UK), then HRP staining kit carrying secondary antibody was used to stain. Sections were imaged by dedicated image microscope.

### Western blotting, enzyme-linked immunosorbent assay, and real-time reverse transcription-quantitative PCR (real-time RT-qPCR)

Western blot was implemented as previously described [19]. Briefly, total proteins were extracted by RIPA Lysis Buffer (Beyotime, Shanghai, China) and quantified using a Pierce BCA Protein Assay Kit (Thermo Fisher Scientific, Waltham, MA, USA). These proteins were separated by 8–10% SDS-PAGE and transferred onto polyvinylidene difluoride membranes (Millipore, Billerica, MA, USA). The target proteins (MSP or RON) were immunoblotted with primary antibodies and then incubated with secondary antibodies coupled with HRP. The blots were photoed and quantitative analyzed using a immunoblotting imaging instrument.

MSP and RON levels in the plasma were estimated by enzyme-linked immunosorbent assay (catalogue no. ab76822 and ab70936); Abcam) according to the manufacturer’s instructions. The lowest detectable MSP and RON concentrations were 8.0 and 8.0 pg/mL, respectively.

Real-time RT-qPCR was performed with the Prime-Script™ RT reagent kit (TaKaRa, Shiga, Japan) and Platinum SYBR Green qPCR SuperMix-UDG (Life Technologies) following the manufacturer’s instructions [20]. Divergent primers, rather than the more commonly used convergent primers, were designed for circCRAMP1L. The primers for circCRAMP1L and glyceraldehyde-3-phosphate dehydrogenase (GAPDH) were synthesised by Landm biotech (Guangzhou, China). The sequences of the GAPDH and circCRAMP1L primers were displayed into additional Table S1. the △Ct method was used for the data analysis. All results are expressed as the mean ± standard deviation of three independent experiments.

### TEV-1 cell culture

TEV-1, a human extravillous trophoblast cell line, was gifted from The University of Hong Kong and The Chinese University of Hong Kong. TEV-1 was cultured in Dulbecco’s modified Eagle’s medium (DMEM)/F12 1:1 medium containing 5% foetal bovine serum according to manufacturer’s instructions [21]. Briefly, stable TEV-1 cells were cultured up to 80% confluence and digested with trypsin. The suspended cells were grown in replaced culture liquid with F12: DMEM, HEPES, streptomycin, and 5% foetal bovine serum (Life Technologies), followed by incubation in 5% CO_2_ air at 37 °C.

### 3-(4,5-dimethylthiazol-2-yl)-2,5-diphenyltetrazolium bromide (MTT) assay

Trophoblast cell proliferation rates were evaluated by MTT assay [22]. The following groups were analysed in this experiment: si-MSP, si-RON, or circCRAMP1L-overexpressing group, and negative control group. TEV-1 cells were transfected with a circCRAMP1L-overexpressing vector or si-RON or si-MSP or empty vector and then seeded into a 96-well plate containing DMEM/F12 medium. On each day for up to five days, MTT solution (1 mg/mL) was added to the wells; after 6 h, 150 μL dimethyl sulfoxide was added. Cell growth was measured at 490 nm on a SpectraMax i3x Microplate Reader (Molecular Devices, Sunnyvale, CA, USA).

### Invasion experiment

Biocoat Matrigel Invasion Chambers (BD Biosciences, Franklin Lakes, NJ, USA) were applied to assess TEV-1 invasion [23]. TEV-1 cells (1 × 10^6^ cells/mL) were inoculated into the Matrigel-covered chambers and incubated in 500 μL culture medium containing 20% serum for forty-eight hour. The invasion rooms were fixed and stained for 10 min and observed in microscopic method to evaluate the amount of invaded cells. To measure invasion changes under different conditions including circCRAMP1L + Si-RON, Si-MSP, or Si-RON.

### RNA immunoprecipitation (RIP)

Binding of circCRAMP1L to MSP protein was detected utilizing the EZ-Magna RIP kit (Millipore) [24]. In accordance with the manufacturer’s protocols, TEV-1 cells were seeded in fifteen centimetres’ cell-plates, gathered with centrifugal method, and splitted into RIP lysed solution with 100 mL of buffer containing the lysates of 1–2 × 10^7^ cells. Cell degradation products were put to use for immunoprecipitation in the company of antibodies against MSP (1:500) or RON (1:100) and normal immunoglobulin G (IgG; 1:100; ab109489, Abcam) and the target RNA was extracted and purified for analysis by RT-qPCR using primers specific for linc00473 and IgG (Supplementary TableS1).

### Double luciferase gene experiment

Double luciferase gene experiment was executed to demonstrate the relevance of circCRAMP1L and MSP [22]. MSP-3′ untranslated region (UTR) (including the seed sequence) cDNA fragments were amplified by PCR and then subcloned downstream of the luciferase genes in the pGL4 plasmid; this plasmid was named as pGL4-MSP-3′UTR-WT. The mutant recombinant plasmids, named as pGL4-MSP-3′UTR-MUT (without objective sequence), were mutated by PCR mutation pattern. circCRAMP1L vectors or scrambled controls and MSP-3′UTR-WT or MSP-3′UTR-MUT were co-transfected into HEK293T cells. The wild-type MSP 3′UTR and mutant type MSP 3′UTR primer sequences are shown in Supplementary Table S2. At 48 h post-transfection, relative luciferase activity was normalised to Renilla luciferase activity and measured with a GloMax 96 Microplate Luminometer (Promega, Madison, WI, USA).

### Statistical analysis

SPSS 24.0 software (SPSS, Inc., Chicago, IL, USA) and MedCalc 11.4. (MedCalc Software bvba, Ostend, Belgium) were used for statistical analyses. The independent sample *t*-test or Mann-Whitney U- test were performed for continuous data comparing and categorical data were analyzed by Fisher’s exact test. All *P* values are two-tailed and *P* <  0.05 were regard as significant.

## Results

**Fig. 1:**
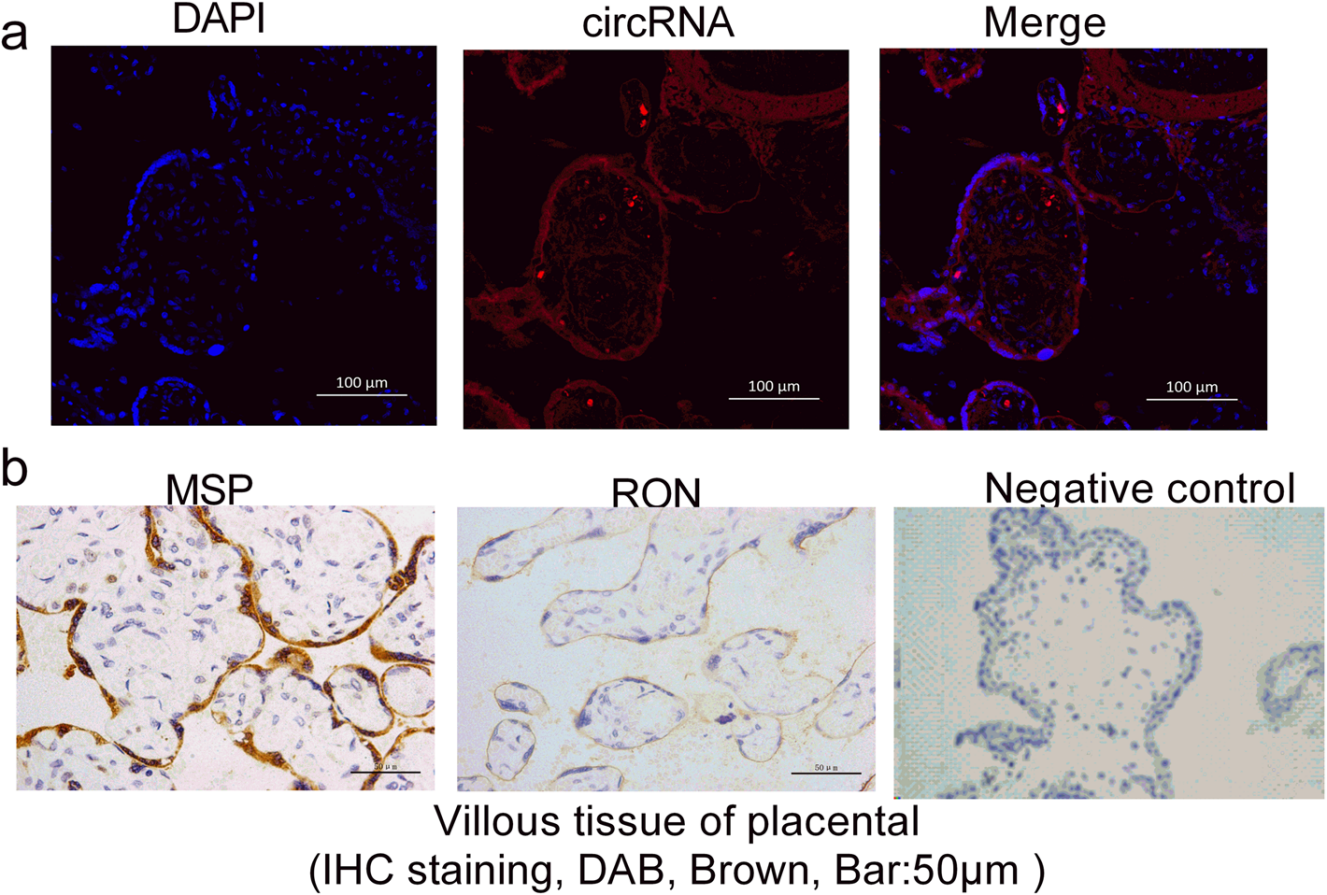
**a** Fluorescent in situ hybridisation results showing the localisation of circCRAMP1L in placenta tissue and **b** immunohistochemistry analysis showing MSP and RON expression in villous tissue of placenta

**Fig. 2:**
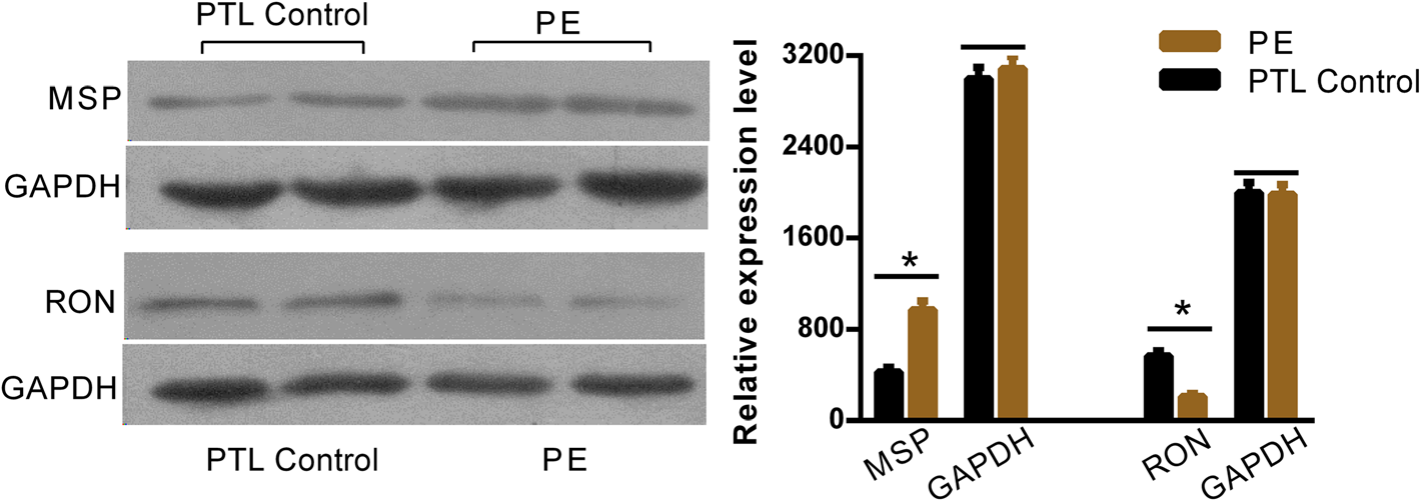
Expression of MSP and RON protein levels determined by western blotting. MSP protein levels in PE placentas were substantially increased compared to in PTL pregnancy placentas, whereas RON protein levels showed the opposite expression patterns (*P < 0.05)

**Fig. 3:**
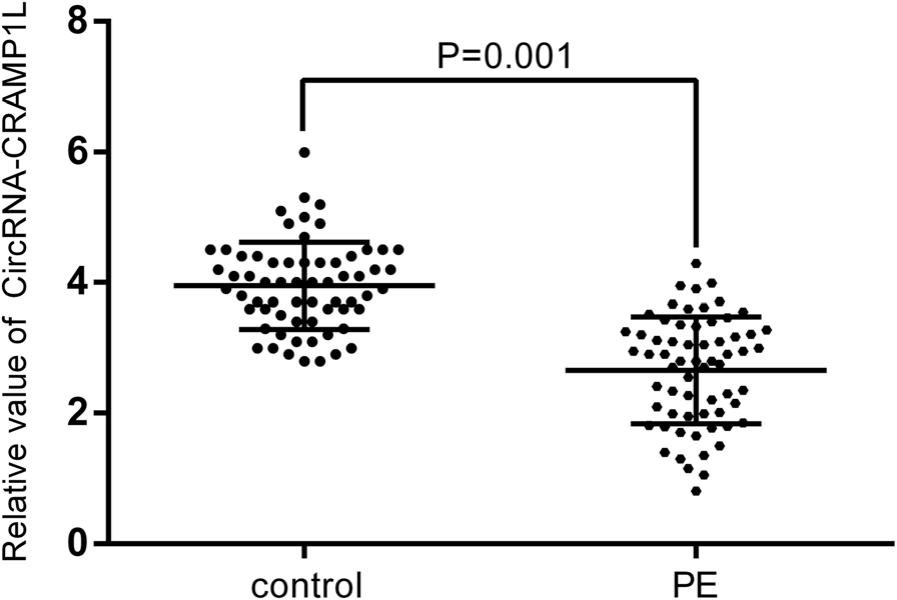
circCRAMP1L expression in circulating plasma of patients with preeclampsia compared to normal pregnant women (2.66 ± 0.82 vs. 3.95 ± 0.67, *P* = 0.001)

**Fig. 4:**
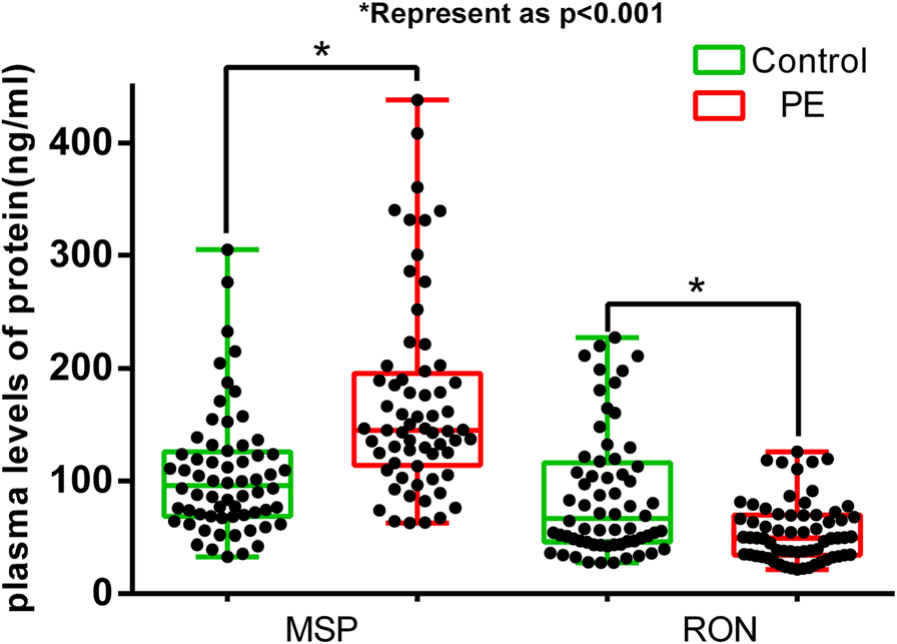
Plasma MSP of PE group were significantly elevated than the control (170.25 ± 42.11 vs. 106.44 ± 38.31, ng/mL, *P* < 0.001). In contrast, the plasma RON in PE group were significantly reduced than the control (55.36 ± 31.25 vs. 87.41 ± 27.48 ng/mL, P < 0.001)

The clinical characteristics of patients with PE, normal pregnancy controls, and PTL pregnancy controls are shown in Table 1 and Table 2. The fluorescent in situ hybridisation array verified the localisation of circCRAMP1L in the placenta tissue of patients with PE, which indicated that placenta circCRAMP1L is related to PE (Fig. 1a). Immunohistochemistry analysis revealed MSP and RON expression in the placental villous tissue (Fig. 1b). The differential expression of MSP and RON protein levels between PE placentas (*n* = 30) and PTL control placentas (n = 30) was validated by western blot assays. As shown in Fig. 2, the MSP and RON protein levels in PE placentas were substantially increased and decreased, respectively, compared to in PTL pregnancy placentas (Fig. 2). Compared to the controls (3.95 ± 0.67, *n* = 64), plasma circCRAMP1L level was significantly decreased in PE (2.66 ± 0.82, n = 64) (*P* <  0.001), confirming that plasma circCRAMP1L derived from the placenta is closely related to PE pathogenesis (Fig. 3). Plasma MSP of PE group were significantly elevated than the control (170.25 ± 42.11 vs. 106.44 ± 38.31, ng/mL, P <  0.001) (Fig. 4). In contrast, the plasma RON in PE group were significantly reduced than the control (55.36 ± 31.25 vs. 87.41 ± 27.48 ng/mL, P <  0.001) (Fig. 4).

**Table 1:** Clinical characteristics of patients with PE and normal pregnancies in plasma study

Characteristic	PE (***n*** = 64)	Control (***n*** = 64)	***P-***value
Maternal Age (year)	32.55 ± 5.24	31.26 ± 4.86	0.170
Pre-pregnancy BMI (kg/m^2^)	23.07 ± 2.14	22.66 ± 1.08	0.085
Gestational age (week)	34.45 ± 3.37	38.23 ± 2.11	< 0.001
Systolic (mmHg)	156.27 ± 14.39	114.51 ± 9.89	< 0.001
Diastolic (mmHg)	102.63 ± 8.51	76.73 ± 8.09	< 0.001
Proteinuria (> 0.3 g/24 h n, %)	100 (100%)	0 (0%)	> 0.05
Smoking (n, %)	0 (0%)	0 (0%)	> 0.05
New-born weight (g)	2459.16 ± 678.24	3466.5 ± 534.28	< 0.001

*PE* preeclampsia, *BMI* body mass index

**Table 2:** Clinical characteristics of patients with PE and PTL control pregnancies in placenta study

Characteristic	PE (***n*** = 30)	Control (***n*** = 30)	***P-***value
Maternal Age (year)	32.23 ± 4.16	31.55 ± 6.24	0.210
Pre-pregnancy BMI (kg/m^2^)	22.92 ± 3.07	22.51 ± 2.16	0.079
Gestational age (week)	34.61 ± 3.83	34.23 ± 2.11	0.470
Systolic (mmHg)	155.66 ± 15.54	118.72 ± 8.72	< 0.05
Diastolic (mmHg)	103.01 ± 7.73	79.66 ± 3.91	< 0.05
Proteinuria (> 0.3 g/24 h, n, %)	100 (100%)	0 (0%)	> 0.05
Smoking (n, %)	0 (0%)	0 (0%)	> 0.05
New-born weight (g)	2433.75 ± 538.19	2512.7 ± 681.46	0.088

*PE* preeclampsia, *BMI* body mass index, *PTL* unexplained preterm labour

**Fig. 5:**
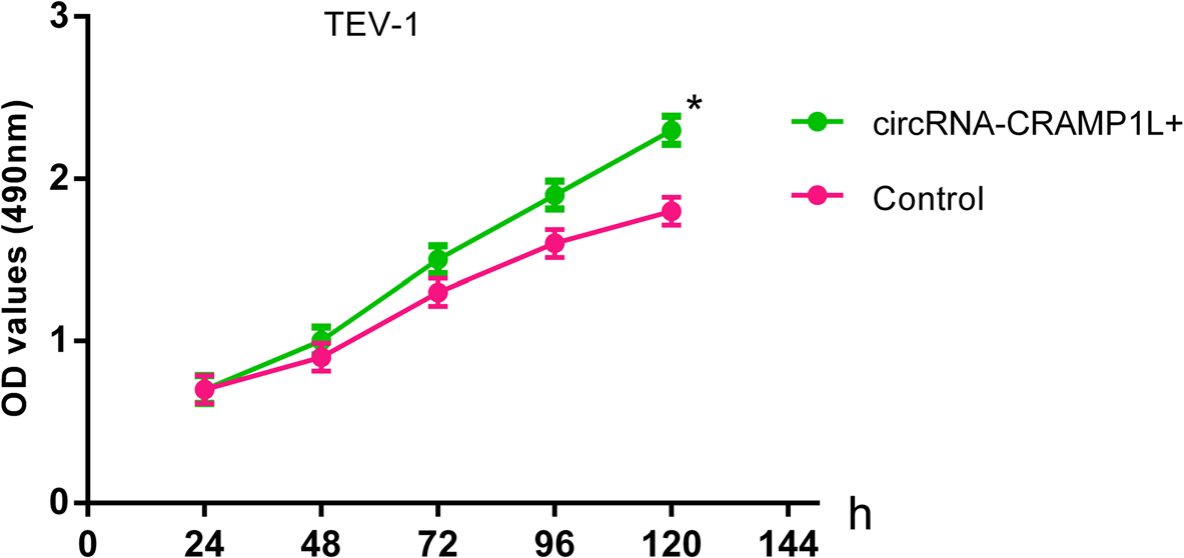
Regulatory effects circCRAMP1L on trophoblast cell proliferation. Compared to the control group, cell proliferation was highly facilitated by circCRAMP1L overexpression (* compared with the control and *P* < 0.01)

**Fig. 6:**
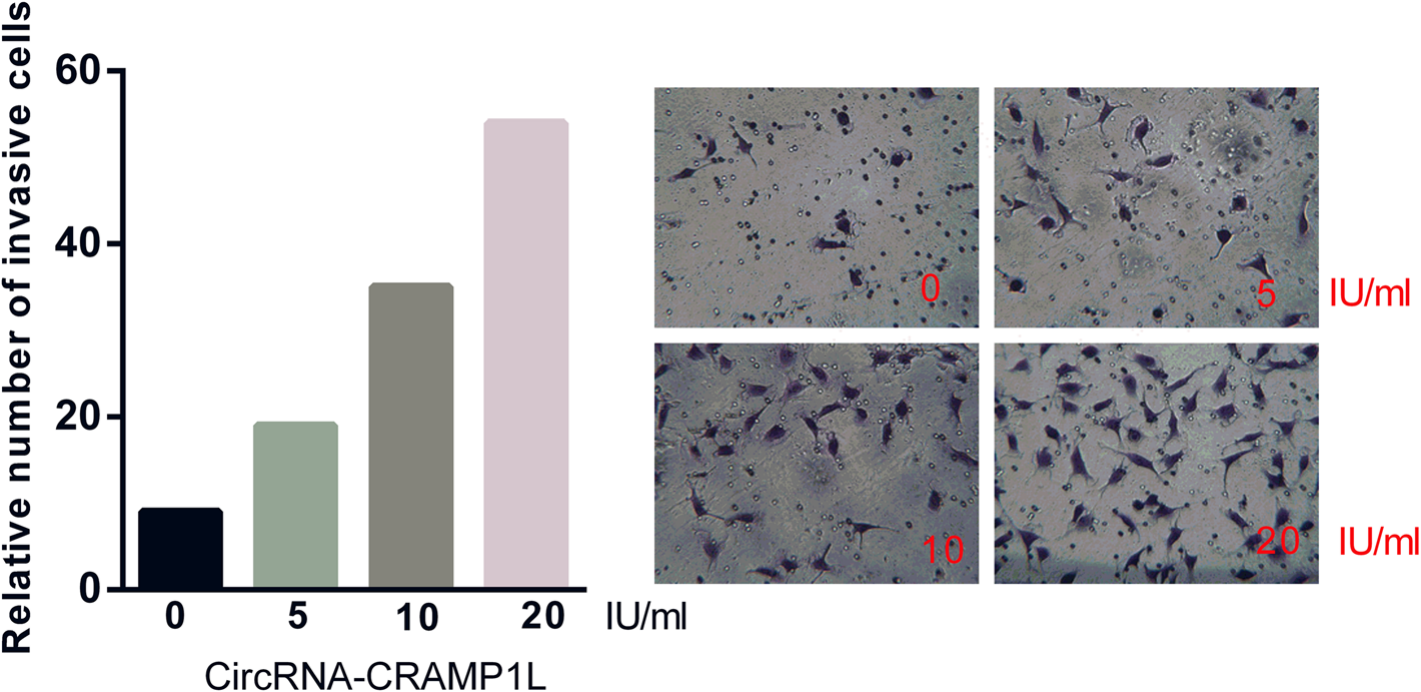
Regulatory effects circCRAMP1L on trophoblast cell invasion. Invasion of trophoblast cells, which were TEV-1 cells treated with siRNA-RON, proportionally increased with the expression level of circCRAMP1L

**Fig. 7:**
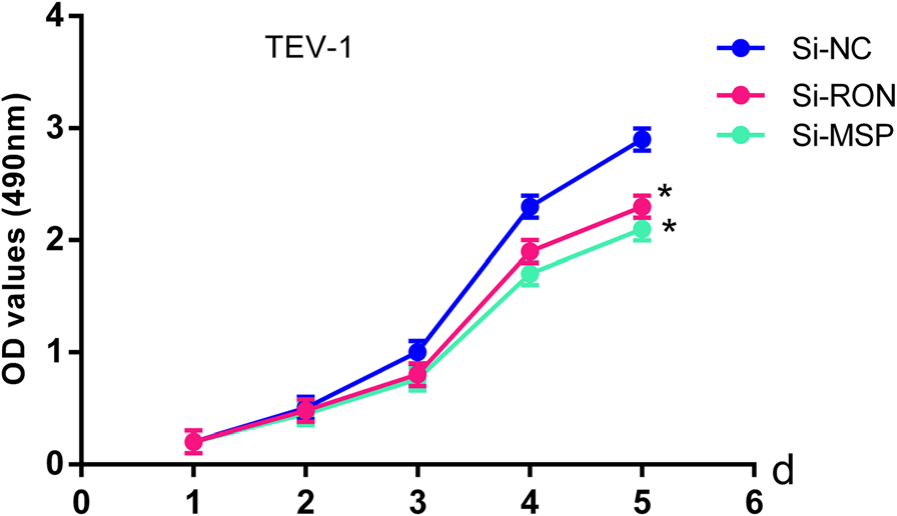
Cell proliferation in siRNA-RON and siRNA-MSP groups was slower than in the siRNA-NC groups (* compared with the si-NC and P < 0.01)

**Fig. 8:**
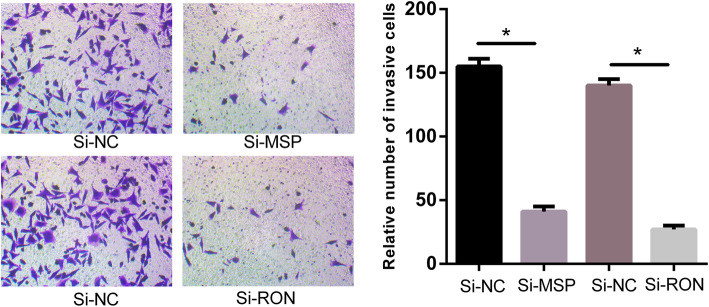
TEV-1 Cell invasion capacities variation when siRNA-RON or siRNA-MSP. Cell invasion in the siRNA-RON group (41.30 ± 4.83 cells) was inhibited compared to in the siRNA-NC group (155.10 ± 6.72 cells, *P < 0.01). Similarly, cell invasion in the siRNA-MSP group (140.21 ± 5.55) significantly differed from that in the siRNA-NC group (27.62 ± 3.48) (*P < 0.01)

To provide insight into the biological function of circCRAMP1L in PE pathogenesis, MTT proliferation and transwell invasion assays were carried out. Compared to the control group, cell proliferation (Fig. 5) was greatly increased by circCRAMP1L overexpression; furthermore, the invasion of trophoblast cells, which were siRNA-RON TEV-1 cells, increased proportionally with the expression level of circCRAMP1L (Fig. 6); in contrast, cell proliferation in the siRNA-RON group and siRNA-MSP group was lessened than the siRNA-NC groups (*P* <  0.01). In the siRNA-RON group (41.30 ± 4.83 cells), cell invasion was inhibited compared to in the siRNA-NC group (155.10 ± 6.72 cells, P <  0.01) (Fig. 7), Similarly, cell invasion in the siRNA-MSP group (140.21 ± 5.55) significantly differed from that in the siRNA-NC group (27.62 ± 3.48) (P <  0.01) (Fig. 8).

**Fig. 9:**
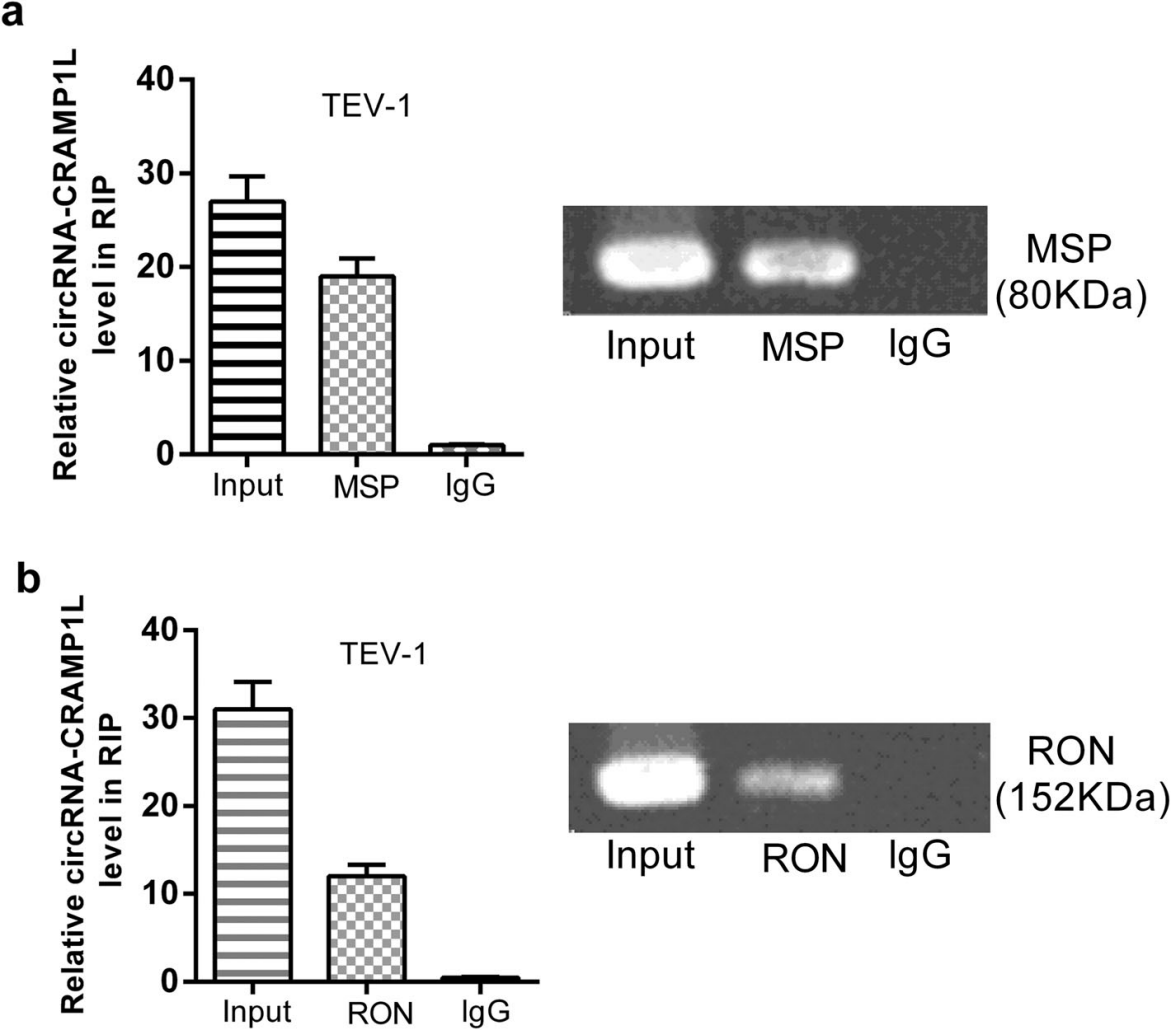
RNA immunoprecipitation assay of the interaction of circCRAMP1L with MSP /RON in TEV-1 cells

**Fig. 10:**
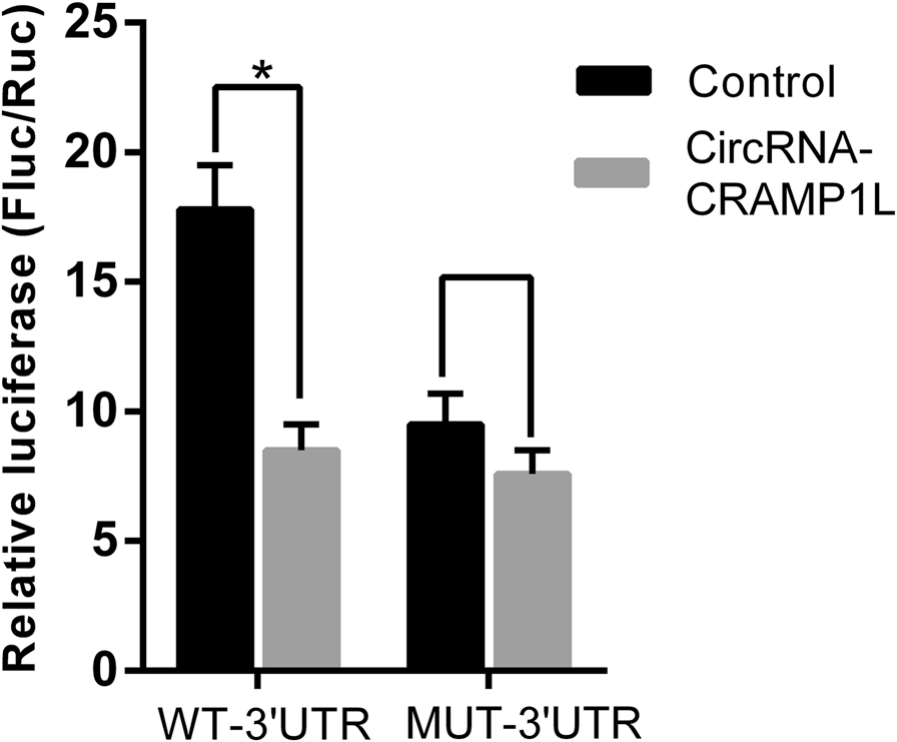
Dual-luciferase assay of the interaction of circCRAMP1L with MSP. Compared to the negative control, circCRAMP1L significantly inhibited the activity of the wild-type, but not the mutant, human MSP 3′UTR reporter gene (*P < 0.05)

To further clarify the mechanism of circCRAMP1L, we performed RIP and dual-luciferase reporter assays using TEV-1 cells. The results indicated that circCRAMP1L mediates the regulation of cell proliferation and invasion via MSP and RON proteins, both which are closely related to the occurrence and development of PE (Figs. 9 and 10).

**Fig. 11:**
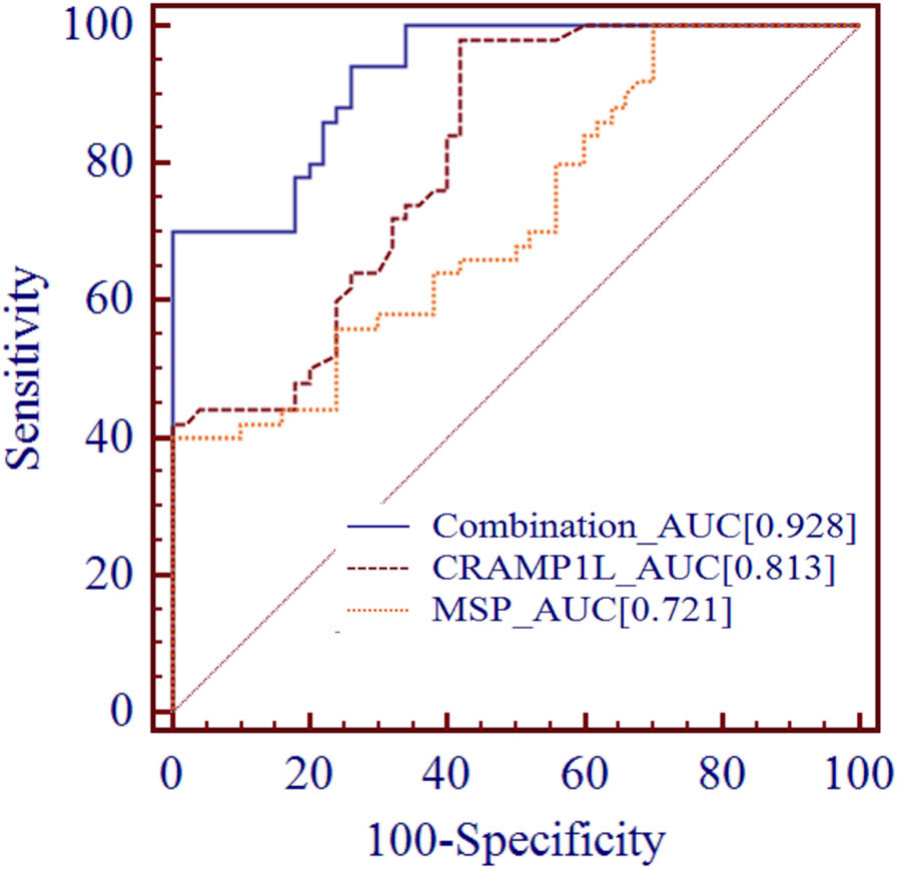
Receiver operating characteristic curve analysis. As a single predictor, MSP or circCRAMP1L showed low efficiency. When MSP was utilised in combination with circCRAMP1L, the predictive performance increased [AUC = 0.928 (95%CI 0.882–0.974)], with 80.0% sensitivity and 80.0% specificity

The area under the receiver operating characteristic curve (AUC) of circCRAMP1L was 0.813 (95% confidence interval [CI] 0.731–0.895); its sensitivity and specificity were 64.0 and 74.0%, respectively, and the cut-off value was 10.3 (cycle threshold value). However, taking the plasma MSP factor as a single predictor resulted in a sensitivity of 64%, specificity of 62%, and AUC of 0.721 (95% CI 0.622–0.819) in PE. While MSP was utilised in conjunction with circCRAMP1L, the predictive performance of the AUC was up to 0.928 (95% CI 0.882–0.974), with 80.0% sensitivity and 80.0% specificity (Fig. 11).

## Discussion

PE is a complex disease for gravida and one of the leading causes of pregnancy-associated morbidity and mortality. The management of this syndrome requires improvement [25]. Reportedly, PE has a long-term impact on cardiovascular disease development [26]; hence, early detection and intervention are particularly important. Recently, increasing evidence has suggested that circRNA is associated with PE [27–29].

In the study, we found that circCRAMP1L was present in the placenta tissue of patients with PE. Bioinformatics prediction indicated that MSP is the target protein gene of circCRAMP1L. Furthermore, protein function analysis revealed that the MSP-RON axis may be involved in PE development. The role of MSP-RON in the growth and metastasis of tumours has already been reported extensively.

Some reports have shown that RON may be participated in the placental embedment [30, 31]. We also observed that MSP and RON are differentially expressed in PE and control placenta and may be related to PE pathogenesis, which is consistent with our previous study [31].

Further, circCRAMP1L was obviously decreased in the circulating plasma of patients with PE compared to in controls. We performed used biological prediction and molecular experiments to verify that circCRAMP1L is involved in trophoblast cell proliferation and invasion via the MSP-RON axis in PE.

The MSP-RON axis is essential for placental development; previous animal experiments showed that foetal mice lacking RON were more likely to die from placental development problems [30]. MSP is the only ligand of RON, and MSP may be involved in placenta development. Analogously, we found that the placental cell proliferation and invasion capacity was lower when RON or MSP was knocked down (shRNA RON or MSP). It is well-known that placental development and placental trophoblast invasion are closely related to the pathogenesis of PE. MSP has been reported to promote the invasion of a variety of cells. However, we found that MSP was increased in the placental tissue of patients with PE, leading to decreased invasiveness. Literature review revealed that MSP can be divided into active and inactive types [32]. The decrease in circCRAMP1L may have inhibited activation of MSP and led to a decrease in RON. However, the increasing MSP detected in the placenta and plasma may have inactive. This would explain why MSP was increased whereas RON was decrease. Further experiments are needed to be clarify this point.

Through this preliminary study, we confirmed that reduced circCRAMP1L influenced the physiological function of trophoblast cells via MSP/RON axis and that circCRAMP1L/MSP/RON may play a role in PE pathogenesis. Additionally, circCRAMP1L, together with the downstream protein factor MSP, may heighten the ability to predict PE.

There are two limitations in the study. Smaller sample size was first restriction, and thus we did not classify the different types of PE. Second, we only analysed the total increase in MSP. Further studies are needed to distinguish active from inactive MSP and determine how circCRAMP1L affects MSP activation.

## Conclusions

In conclusion, circCRAMP1L may be beneficial for preventing and treating PE and may be a novel biomarker for PE. Further studies of the specific and downstream mechanisms are needed, and the efficacy of using more predictive factors jointly should be examined.

## Supplementary information

**Additional file 1: Supplementary Table S1.** Primer sets used for RT-PCR, qRT-PCR, and RNA immunoprecipitation (RIP). **Supplementary Table S2.** Primer sets used for Dual-luciferase reporter assay.

**Additional file 2: Fig. S1.** Original images of western blot (the cropping blot images correspond to fig2 in manuscript, the samples derive from the same experiment and that blots were processed in parallel.). **Fig. S2.** Original images of RNA immunoprecipitation(RIP) experiment (the agarose gel images correspond to fig9 in manuscript).

## Data Availability

The detailed datasets used and analysed during the current study are available from the corresponding author on reasonable request.

## Abbreviations

circRNAs: circular RNAs
circCRAMP1L: circRNA-CRAMP1L
MTT: 3-(4,5-dimethylthiazol-2-yl)-2,5-diphenyltetrazolium bromide
RIP: RNA immunoprecipitation
TEV-1: Human first-trimester extravillous trophoblast cell line
AUC: Area under the receiver operating characteristic curve
MSP: Macrophage stimulating protein
RON: R*ecepteur d′origine nantais*, receptor of macrophage stimulating protein
PE: Preeclampsia
PTL: Unexplained preterm labour
BMI: Body mass index
EDTA: Ethylenediaminetetraacetic acid
FISH: Fluorescent in situ hybridisation
DAPI: 4’, 6-diamidino-2-phenylindole
GAPDH: Glyceraldehyde-3-phosphate dehydrogenase
DIG: Digoxigenin
RT-qPCR: Reverse transcription-quantitative polymerase chain reaction
SDS-PAGE: Sodium dodecyl sulfate-polyacrylamide gel electrophoresis
HRP: Horseradish peroxidase
SYBR: A fluorescent dye
DMEM: Dulbecco’s modified Eagle’s medium
HEPES: 2-[4-(2-hydroxyethyl)piperazin-1-yl]ethanesulfonic acid
DMSO: Dimethylsulfoxide
IgG: Immunoglobulin G
MUT: Mutant
UTR: Untranslated region
GO: Gene Ontology
IOD [Ave]: Accumulated optical density (average)
ROC: Receiver operating characteristic
CT: Cycle threshold
