# Supplementary material for: circCRAMP1L is a novel biomarker of preeclampsia risk and may play a role in preeclampsia pathogenesis via regulation of the MSP/RON axis in trophoblasts
Source: BMC Pregnancy Childbirth. 2020 Oct 27;20:652. doi: 10.1186/s12884-020-03345-5 (PMC7590488; doi:10.1186/s12884-020-03345-5)
Supplement: Supplementary file 1 — Additional file 1: Supplementary Table S1. Primer sets used for RT-PCR, qRT-PCR, and RNA immunoprecipitation (RIP). Supplementary Table S2. Primer sets used for Dual-luciferase reporter assay. [file 12884_2020_3345_MOESM1_ESM.docx]

**Supplementary Table S1.** Primer sets used for RT-PCR, qRT-PCR, and RNA immunoprecipitation (RIP)

| **Primer set** | **Primers** | **Sequence** | **Product size (bp)** | **Application** |
| --- | --- | --- | --- | --- |
| CircRNA- CRAMP1L | Forward | 5′-GACCAGGGCAGCTGACTCGAAG-3′ | 256 | RT-PCR, qRT-PCR,  RIP |
|  | Reverse | 5′-GACATGCTCCACTCATCCGACACC-3′' |  |  |
| convergent (GAPDH) | Forward | 5′-CACTGAGCAAGAGAGGCCCTAT-3′ | - |  |
|  | Reverse | 5′-GCAGCGAACTTTATTGATGGTATT-3′ |  |  |

**Supplementary Table S2.** Primer sets used for **Dual-luciferase reporter assay**

| **Primer set** | **Primers** | **Sequence** | **Product size (bp)** | **Application** |
| --- | --- | --- | --- | --- |
| wild type MSP 3'UTR | Forward | 5'CCACTCGAG GCCCAGCCTTGATGCCATATG 3' | 100 | PCR, |
|  | Reverse | 5'ATCCAATGCGGCCGCTTTTTTACAGGCATAAAGAGGAAAC 3' |  |  |
| Mutant type  MSP 3'UTR | Forward | 5'CAGACATAGGATTGCGTTTCCTCTTTATGCCTGT 3' | 100 |  |
|  | Reverse | 5'AAGAGGAAACGCAATCCTATGTCTGACAAGAAGTT 3' |  |  |

Mutation PCR system

| Mutation PCR system |  |
| --- | --- |
| 2.5 mM dNTP mixture | 2 µl |
| 10×Pyrobest buffer | 2.5 µl |
| MSP 3’UTR-100 (diluted 100-fold) | 1 µl |
| Primers mutMSP 3’UTR-F (5pmol) | 2.5 µl |
| Primers mutMSP 3’UTR-R (5pmol) | 2.5 µl |
| Pyrobest TM DNAPolymerase | 0.3 µl |
| ddH2O | 14.2 µl |
| Total | 25 µl |
| 2.5 mM dNTP mixture | 2 µl |
| 10×Pyrobest buffer | 2.5 µl |
| MSP 3’UTR-100 (diluted 100-fold) | 1 µl |
| Primers mutMSP 3’UTR-F (5pmol) | 2.5 µl |
| Primers mutMSP 3’UTR-R (5pmol) | 2.5 µl |
| Pyrobest TM DNAPolymerase | 0 µl |
| ddH2O | 14.5 µl |
| Total | 25 µl |

Pyrobest TM DNA Polymerase from TaKaRa company. catalog number: DR005A

Mutation Amplification conditions: (1)95℃ 5 min,(2)18 Cycle

95℃ 30 sec

55℃ 30 sec

68℃ 8 min

68℃ 5 min

0℃ hold

Then ➀Amplification, ➁electrophoresis, ➂digestion with 1µl DpnI ( Promega company, catalog number r6231 ), ➃After enzyme digestion, the product was transfection into E. coli, and ➄then pick out monoclonal bacteria , ➅Finally  the mutant genes were identified by sequencing.
