# Supplementary material for: circCRAMP1L is a novel biomarker of preeclampsia risk and may play a role in preeclampsia pathogenesis via regulation of the MSP/RON axis in trophoblasts
Source: BMC Pregnancy Childbirth. 2020 Oct 27;20:652. doi: 10.1186/s12884-020-03345-5 (PMC7590488; doi:10.1186/s12884-020-03345-5)
Supplement: Supplementary file 2 — Additional file 2: Fig. S1. Original images of western blot (the cropping blot images correspond to fig2 in manuscript, the samples derive from the same experiment and that blots were processed in parallel.). Fig. S2. Original images of RNA immunoprecipitation(RIP) experiment (the agarose gel images correspond to fig9 in manuscript). [file 12884_2020_3345_MOESM2_ESM.doc]

**
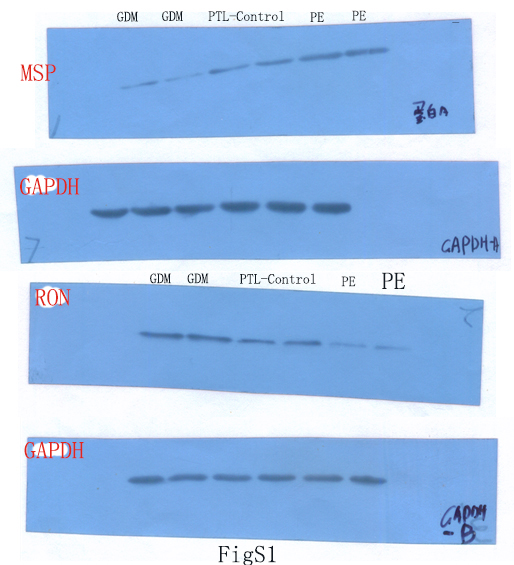
**

**FigS1:** Original images of western blot (the cropping blot images correspond to fig2 in manuscript, the samples derive from the same experiment and that blots were processed in parallel.)


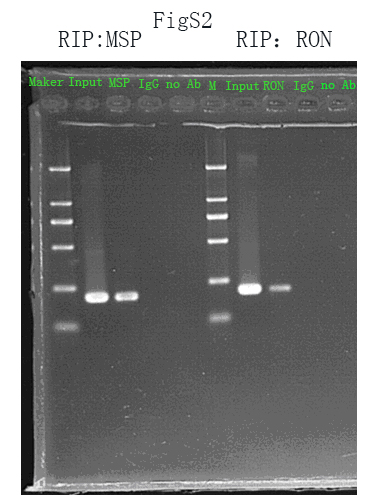


**FigS2:** Original images of RNA immunoprecipitation(RIP) experiment (the agarose gel images correspond to fig9 in manuscript)
